# The emergence of COVID-19 in the Democratic Republic of Congo: Community knowledge, attitudes, and practices in Kinshasa

**DOI:** 10.1371/journal.pone.0265538

**Published:** 2022-06-21

**Authors:** Pierre Z. Akilimali, Désiré K. Mashinda, Aimé M. Lulebo, Eric M. Mafuta, Marie A. Onyamboko, Nguyen Toan Tran

**Affiliations:** 1 School of Public Health, University of Kinshasa, Kinshasa, Democratic Republic of Congo; 2 Australian Centre for Public and Population Health Research, Faculty of Health, University of Technology Sydney, Sydney, NSW, Australia; 3 Faculty of Medicine, University of Geneva, Geneva, Switzerland; VIT University, INDIA

## Abstract

**Background:**

The first COVID-19 case in the Democratic Republic of Congo (DRC) was reported on 10 March 2020 in Kinshasa, prompting the government to promote internationally agreed non-pharmacological interventions for infection prevention and control. Public compliance to these measures is critical and depends on the knowledge, attitudes, and practices (KAP) of communities regarding COVID-19, for which there was no data. This study aimed to bridge that gap.

**Methods:**

A community-based cross-sectional study was conducted in Kinshasa in June 2020, during the emergency state, following a four-stage sampling process. Master’s students from the Kinshasa School of Public Health conducted the survey. Descriptive and regression analyses were performed.

**Results:**

The study enrolled 726 women and 600 men (mean age: 43; SD 16-85). Nearly everyone heard about COVID-19 (mainly through television, radio, and street reports), but only 17% were highly knowledgeable about its transmission modes, signs and symptoms, and preventive measures. More than 80% of participants believed in the disease’s seriousness; however, only 21% found the total lockdown acceptable. Nonetheless, 86% reported regular hand cleaning and mask-wearing followed by physical distancing (72%). Poorer, younger, and non-Catholic participants were overall markedly less knowledgeable and had comparatively lower levels of health-protective attitudes, acceptance, and practices. The education level and household size did not matter. Female participants tended to show fewer enabling attitudes and practices toward COVID-19 prevention measures compared to men.

**Conclusion:**

Adequate public health information to improve the population’s KAP related to COVID-19 is critical and must be designed with and delivered to the community—considering the specific needs of diverse sub-groups and contexts. Studies in Kinshasa and similar settings are necessary to understand the barriers to and enablers of acquiring, applying, and maintaining the optimal population’s KAP for COVID-19 prevention and control.

## Introduction

The first case of the Coronavirus Disease 2019 (COVID-19) pandemic in the Democratic Republic of Congo (DRC) was registered in its capital Kinshasa on 10 March 2020. As of 4 June 2021, almost 15 months later, the country recorded a cumulative number of 786 deaths out of 31,934 confirmed cases [1]. The outbreak has spread to 23 out of the 26 national provinces, but Kinshasa remains the most affected part of the country, accounting for approximately a third of all cases. The national COVID-19 Stringency Index, a composite measure including travel bans, school closures, workplace closures, and gathering restrictions, among others, was still at 0 (no governmental measures) out of 100 (strictest measures) until 19 February 2020 [2]. A day later, it slowly climbed to reach 11 on 17 March before increasing sharply to 81. The Index plateaued at that level for over four months and has been oscillating between 59 and 19 between 26 July 2020 and 4 June 2021.

Due to recurrent financial difficulties, the DRC has not been able to carry out systematic mass screening of its entire population [3]. Voluntary screening and testing of suspect individuals and their contacts and, if positive, isolation have been the primary strategy. Therefore, from the start of the outbreak, its prevention and control depended mainly on the level of compliance by the population to the non-pharmacological prevention measures recommended by the Ministry of Health (MOH) based on the guidance of the World Health Organization (WHO), namely physical distancing, wearing a mask, avoiding crowds, cleaning hands, avoiding touching nose, mouth, and eyes with uncleaned hands, and coughing into a bent elbow or single-use tissue [4]. Respecting such measures rely on the level of knowledge of the population about the infection, the perception of the risk of contracting it, and specific barriers that the public may face in applying protective steps. Therefore, the knowledge, attitudes, and practices (KAP) toward COVID-19 are critical in determining the population’s readiness in accepting and enacting recommended behavioral change measures. Assessing COVID-19 related KAP in the general public was deemed necessary from the early phase of the outbreak to provide baseline information and offer insights into developing preventive strategies and health promotion programs to address inadequate understanding, perspectives, and behaviors [5].

In response to the emergence of the pandemic in Africa, several Sub-Saharan countries conducted such KAP surveys from late March to May 2020. For example, in Ethiopia, a study done in its capital Addis Ababa found that 59% of the public had moderate knowledge about COVID-19, and 37% had good knowledge. Around 60% of them had a positive attitude towards preventive measures and good practices to mitigate the pandemic [6].

In Uganda, in a population sample with three-quarters of participants from urban settings and with tertiary or university levels of education, 94% were found to be knowledgeable about COVID-19 [7]. Around half (51%) had positive attitudes towards the presidential directives and MOH guidelines and adhered to practicing public health preventive measures, although these were significantly better observed by women (56%) than men (43%). In Sudan, an online survey determined that over 90% knew that the COVID-19 is transmitted through droplets and had a positive attitude toward the COVID-19 pandemic [8]. However, only 49% of the participants had a good practice toward infection prevention and control—sound knowledge was associated with enacting a good practice. Also targeting educated participants with Internet access, a survey in Nigeria found that 78% exhibited good knowledge of COVID-19, which positively affected their attitude and practice grades [9].

In southeastern DRC, a study was done in April 2020 in public markets in the cities of Kolwezi, Likasi, Lubumbashi, and Lwambo, showing that only 30% of participants had correct knowledge of COVID-19, 88% had no confidence in the government’s ability to manage the pandemic, and 98% were concerned about the resulting increase in food insecurity [10].

This KAP research conducted in June 2020 in Kinshasa aimed at determining the level of knowledge, attitudes, and practices of the community regarding COVID-19, including their acceptability of government recommendations.

## Materials and methods

### Settings

The DRC is the fourth most populous African country. The capital of the DRC, Kinshasa, is one of the world’s “megacities” [11]. With over 11 million people, Kinshasa is Africa’s third-largest city (after Lagos and Cairo) and one of the continent’s most rapidly growing urban areas. Kinshasa is divided into 35 Health Zones, with each Health Zone subdivided into Health Areas. Only around half of the population has access to water and a third to basic sanitation. Lack of access to water and sanitation, coupled with poor hygiene behaviors, malnutrition, and food insecurity, are among the top five risk factors associated with death and disability in the country [12].

### Study design and sampling

This was a quantitative cross-sectional study conducted in Kinshasa from 9-13 June 2020. The sample size was calculated to correctly assess the proportion of acceptability of government measures and KAP among households living in Kinshasa. We used a population proportion of 50% since no data were available when the study was designed. Applying the Kish-Leslie formula for power calculation with a 5% margin of error and a confidence interval (CI) of 95%, the minimum required sample size was 384. We added 10% for contingency, and a total of 423 individuals were invited to participate in the study per Heath Zone.

Applying a multi-stage sampling strategy and informed by budgetary constraints, geographical accessibility, and safety considerations for data collection, we conveniently selected three Health Zones in the first stage (Lemba, Matete, and Mont Ngafula, the latter being the poorest among all three). Second, three Health Areas were randomly selected in each Health Zone. Third, three streets were randomly selected in each Health Area. Finally, households were selected using a systematic door-to-door approach after listing all households in each street. In each household, the household head or spouse was interviewed.

### Measures

The KAP survey consisted of five sections: socio-demographic information, knowledge about COVID-19, attitudes toward COVID-19, including the acceptability of related governmental decisions, and practices relevant to COVID-19. Socio-demographic information included gender, age, religion, education level, income level, household size, and whether housing was in informal settlements (slums). The level of education was considered “low” if high school education or vocational training was not completed, “medium” if these were completed, and “high” if higher education or university were completed. The socio-economic level was determined by a wealth index constructed on a set of household assets (radio, tape recorder, television set, bicycle, hand torch, horse or donkey cart), housing conditions (roof material, number of rooms, wall type, windows, availability, and type of latrine), and ownership of domestic animals. The study participants were ranked according to the wealth index score, divided into terciles. The analyses combined the second (middle-income) and third (high-income) terciles.

The level of knowledge was assessed using the following questions: transmission pathways, signs and symptoms, means of prevention, and COVID-19 hotline number. Respondents with a score ≥ 75% of correct answers were considered to have a high level of knowledge. A score between 50-74% corresponded to a medium level and < 50% to a low level.

Attitude-related questions revolved around believes in infectiousness and curability, effectiveness and acceptability of preventive measures, and stigmatization toward COVID-19.

To assess COVID-19 prevention practices, we used and adapted the recommendations by the WHO and the Ministry of Health as well as a validated survey conducted earlier by Taghrir et al. from Iran [13]. The survey had a Cronbach’s alpha for reliability testing of 0.87 for the pilot phase and 0.80 for the full study. The Cronbach’s alpha for our knowledge, attitudes, and practices surveys were 0.84, 0.87, and 0.74, respectively. We also followed the same scoring system as Taghrir, whereby respondents with a score ≥ 75% of correct answers were considered to have a high level of practice compliance [13]. Survey items included restriction of movement, coughing etiquette, physical distancing, hand hygiene, mask-wearing, avoidance of face-touching with uncleaned hands, avoidance of handshaking or kissing for greeting, and discussion of COVID 19 prevention measures with family or friends.

### Data collection

Using tablets powered by the SurveyCTO application (Dobility, Inc; Cambridge, MA, USA), 85 Master’s students from the Kinshasa School of Public Health collected the data after being trained on the research instruments and ethics and linguistic issues. Each data collector received a set of face masks and hydro-alcoholic gel and was trained on barrier gestures and instructed to apply them in the field. Interviews were conducted in Lingala, the vernacular language in Kinshasa, or French. In consultation with bilingual researchers, we used backward translation to translate items between French and Lingala to ensure linguistic and conceptual equivalence. Data were collected and analyzed anonymously. No personal identifiers of participants were recorded on the survey questionnaire.

### Statistical analysis

After quality control and consistency checks, data were exported into Stata 14 (StataCorp, College Station, TX) for analysis. Descriptive statistics were used to describe the basic characteristics of the study data. Means and standard deviations (SDs) were calculated for normally distributed continuous variables, while proportions with their 95% CIs were calculated for categorical variables. The Z-test was used to compare the proportion between two groups. Pearson’s chi-square test or the Fisher’s exact test were used to test for associations between dependent variables and each independent variable. A logistic regression model was used to identify factors associated with best knowledge, best attitudes, best acceptability of governmental decisions, and best practices—“best” being defined as a score ≥ 75%—and obtain adjusted odds ratio (aOR) and 95% CIs. The final logistic regression model included age, gender, educational status, socioeconomic status, household size, and slum. Variance-inflation factors (VIF) were estimated to assess multicollinearity. A significance threshold of α = 0.05 was used for all tests.

### Ethical approval

The Ethics Committee of the University of Kinshasa approved the study protocol on 3 June 2020 (ESP/CE/020B/2020). As some participants were illiterate and in order to standardize the informed consent process, oral informed consent with a third-party witness was obtained from each participant who was notified that participation was voluntary, and they could withdraw at any time without consequences. A copy of the consent form signed by the witness was given to the participant to keep. Participants had no direct benefits from participating in the study but understood that the results would enable the Government to implement evidence-based interventions for pandemic prevention and control.

## Results

### Characteristics of participants

Table 1 presents the socio-demographic profiles of the 1327 respondents out of 1375 who were invited to participate in the survey (participation rate = 96.5%). The mean age of participants was 43 years (SD 15, range 16-85). There were slightly more female participants (55%) although 76% of households were headed by men. On average, participants were similarly distributed between the income terciles, a majority had a medium (33%) or high level of education (39%), slightly less than half of the households were in informal settlements (46%) and housed seven or more family members (40%). Membership to the Revival Church was the most common (38%), followed by membership to the Catholic Church, Protestant Church, and other faiths. When stratified by Health Zone, respondents from Mont Ngafula were the most underserved with the highest proportion of low-income tercile (56%) and less than 20 liters of water available per person per day (68%) trailing behind Matete and Lemba residents.

**Table 1:** Baseline characteristics of participants.

	n	(%)
**Gender** (1 missing value)		
Male	600	(45.2)
Female	726	(54.8)
**Age** (39 missing values), mean (SD)	43	(16-85)
≤ 24	125	(9.7)
25-35	339	(26.3)
36-49	418	(32.5)
50-64	287	(22.3)
≥ 65	119	(9.2)
**Religion of household head**		
Catholic	394	(29.7)
Protestant	208	(15.7)
Revival Church	502	(37.8)
Others	223	(16.8)
**Education level**		
Low	368	(27.7)
Medium and high	959	(72.3)
**Income terciles** (5 missing values)		
1st	453	(34.3)
2nd and 3rd	869	(65.7)
**Household size**		
≤ 6	794	(59.8)
≥ 7	533	(40.2)
**Slum household** (6 missing values)		
No	709	(53.7)
Yes	612	(46.3)

### Knowledge

Almost all participants (99.7%) across all three Health Zones had heard about COVID-19. Overall, the primary sources of news were television (88%), radio (54%), reports from the street (46%), followed by social media, family members, friends and colleagues, healthcare providers, newspaper, community health workers, schools or universities, and others, without differences between the Health Zones. Table 2 reports findings on knowledge of COVID-19 signs, symptoms, and preventive measures.

**Table 2:** Knowledge of COVID-19 signs, symptoms, and preventive measures by Health Zone.

		Lemba (n=436)	Matete (n=428)	Mont Ngafula (n=459)	Total (n=1323)	
		(%)	(%)	(%)	(%)	p
How could a person acquire COVID-19?						
	Contact with infected people’s saliva	76.4	61.4	58.2	65.2	***
	Breathing or sneezing	44.3	55.8	42.3	47.3	***
	Direct contact with infected people	81.0	75.9	73.9	76.9	*
	Contact with infected people’s personal objects	58.3	44.2	40.7	47.6	***
What are the major signs and symptoms of COVID-19?						
	Fever	92.2	84.8	81.7	86.2	***
	Cough	89.2	84.6	83.4	85.7	*
	Fatique	26.1	22.4	22.4	23.7	
	Muscle pain	15.6	12.9	16.8	15.1	
	Sore throat	27.3	31.8	24.6	27.8	
	Headache	50.7	45.8	42.5	46.3	*
	Shortness of breath	58.3	59.3	56.0	57.8	
What are the current COVID-19 prevention measures?						
	Avoid contact with infected people	73.9	64.7	71.7	70.1	**
	Physical distancing	84.2	76.6	80.0	80.3	*
	Use tissue when coughing or sneezing	47.5	43.2	38.1	42.9	*
	Use elbow when coughing or sneezing	48.6	38.8	39.2	42.2	**
	Frequent hand cleaning	94.0	87.9	83.9	88.5	***
	Face mask	89.0	86.9	85.0	86.9	
	Avoid touching face with unclean hands	40.4	25.2	33.1	33.0	***
	Avoid handshakes or kissing	69.7	64.5	66.4	66.9	
	Avoid unnecessary travel (stay home)	59.9	45.8	53.2	53.0	***
Knows the free COVID-19 hotline number		46.6	50.7	43.1	46.7	*
Knowledge scores						**
	High	20.2	14.5	15.5	16.7	
	Medium	40.5	36.9	35.9	37.7	
	Low	39.3	48.6	48.6	45.5	

Note.

* p-value < .05

** p-value < .01

*** p-value < .001

Overall, three-quarters of respondents reported that transmission occurred with direct contact with a sick person but only half knew that it was airborne. Almost nine out of ten participants reported fever and cough as COVID-19 symptoms followed by shortness of breath and headaches. Hand cleaning, masks, and physical distancing were cited by more than 80% of respondents while cough etiquette and avoiding touching one’s face were the least reported. Around half knew the COVID-19 hotline numbers. There were in general significant differences in knowledge between the Health Zones with Mont Ngafula residents scoring the worst and Lemba the best. Regression analyses indicate that respondents of the 50-64 age group, Catholic denomination, and second and third-income terciles had the best level of knowledge (Table 3).

**Table 3:** Regression analysis of best COVID-19 knowledge and best attitude, acceptance, and practice levels toward COVID-19 prevention measures.

	Best knowledge level		Best attitude level		Best acceptance level		Best practice level	
	aOR	p	aOR	p	aOR	p	aOR	p
**Gender**								
Male	1		1		1		1	
Female	0.94	0.695	0.71	**0.011**	0.81	0.162	1.14	0.295
**Age**								
≤ 24	1		1		1		1	
25-35	1.61	0.142	1.45	0.092	1.76	0.075	1.44	0.100
36-49	1.61	0.133	1.69	**0.015**	2.10	**0.016**	1.41	0.114
50-64	1.85	**0.060**	2.27	**0.001**	2.22	**0.012**	1.41	0.129
≥ 65	1.24	0.584	1.58	0.113	3.67	**<0.001**	1.13	0.657
**Religion of household head**								
Catholic	1		1		1		1	
Protestant	0.50	**0.004**	0.85	0.449	0.62	**0.034**	0.84	0.355
Revival Church	0.57	**0.002**	0.64	**0.006**	0.88	**0.457**	0.80	0.133
Others	0.49	**0.004**	0.78	0.207	0.72	**0.139**	0.85	0.388
**Education level**								
Low	1		1		1		1	
Medium and high	1.34	0.153	1.05	0.764	0.83	0.280	1.14	0.377
**Income terciles**								
1st	1		1		1		1	
2nd and 3rd	1.54	**0.026**	1.42	**0.016**	1.12	0.513	1.64	**0.001**
**Household size**								
≤ 6	1		1		1		1	
≥ 7	0.75	0.070	0.83	0.140	0.92	0.568	0.82	0.106
**Slum household**								
No	1		1		1		1	
Yes	0.88	0.439	0.99	0.983	0.98	0.873	1.20	0.142

Note. significant p-values are in bold; aOR: adjusted odds ratio. Variance inflation factor = 2.22 for all regressions.

### COVID-19 related attitudes and acceptability of governmental decisions

More than 80% of participants believed in the seriousness of COVID-19 (Table 4). More than half of all respondents believed that they could be infected by COVID-19, particularly among men, older age groups, those with higher education and income, and Catholics. Around three-quarters of all participants (with marked differences between religious affiliations) were afraid of getting infected. Around nine out of ten participants believed that one could recover from the disease and three out of four that healthy-looking individuals could transmit the disease. That proportion was significantly lower among women, younger age groups, those with low education and income levels, and those dwelling in informal settlements. Three-quarters of all respondents believed that prevention measures could stop disease transmission, especially among men. Regression analyses show that men and those of the 36-64 age bracket and second and third-income terciles had the best attitude level.

**Table 4:** COVID-19 related attitudes and believes according to baseline characteristics.

	Believes COVID-19 is serious or very serious		Believes one can be infected		Is afraid of getting infected		Believes one can recover from COVID-19		Believes prevention measures can stop transmission		Believes a healthy-looking person can carry the virus		Knows a close family member or friend infected or dead due to COVID-19		Believes an infected family member should not be a secret		Best level of attitudes toward COVID-19 prevention	
	n (%)	p	n (%)	p	n (%)	p	n (%)	p	n (%)	p	n (%)	p	n (%)	p	n (%)	p	n (%)	p
**Gender**				***				*		*		***						***
Male	508 (84.7)		458 (76.3)		437 (72.8)		558 (93.0)		480 (80.0)		488 (81.3)		70 (11.7)		445 (74.2)		459 (76.5)	
Female	609 (83.9)		424 (58.4)		544 (74.8)		652 (89.8)		548 (75.5)		492 (67.8)		75 (10.3)		550 (75.8)		489 (67.4)	
**Age**				***				**				**						**
≤ 24	105 (84.0)		60 (48.0)		87 (69.6)		104 (83.2)		98 (78.4)		79 (63.2)		10 (8.0)		93 (74.4)		75 (60.0)	
25-35	282 (83.2)		210 (61.9)		249 (73.5)		304 (89.7)		257 (75.8)		250 (73.7)		31 (9.1)		246 (72.6)		233 (68.7)	
36-49	350 (83.7)		287 (68.7)		306 (73.2)		392 (93.8)		320 (76.6)		328 (78.5)		50 (12.0)		307 (73.4)		302 (72.2)	
50-64	246 (85.7)		213 (74.2)		221 (77.0)		270 (94.1)		234 (81.5)		221 (77.0)		38 (13.2)		229 (79.9)		224 (78.0)	
≥ 65	102 (85.7)		87 (73.1)		90 (75.6)		106 (89.1)		91 (76.5)		82 (68.9)		13 (10.9)		88 (73.9)		87 (73.1)	
**Religion of household head**				***		*												***
Catholic	340 (86.3)		303 (76.9)		315 (79.9)		364 (92.4)		310 (78.7)		292 (74.1)		55 (14.0)		294 (74.6)		309 (78.4)	
Protestant	172 (82.7)		145 (69.7)		153 (73.6)		193 (92.8)		163 (78.4)		158 (76.0)		19 (9.1)		164 (78.8)		153 (73.6)	
Revival Church	418 (83.3)		294 (58.6)		352 (70.1)		454 (90.4)		386 (76.9)		374 (74.5)		52 (10.4)		372 (74.1)		330 (65.7)	
Others	188 (84.3)		141 (63.2)		162 (72.6)		200 (89.7)		170 (76.2)		156 (70.0)		19 (8.5)		166 (74.4)		157 (70.4)	
**Education level**				***				**				***		***				
Low	312 (84.8)		216 (58.7)		283 (76.9)		323 (87.8)		279 (75.8)		212 (57.6)		16 (4.3)		277 (75.3)		249 (67.7)	
Medium and high	806 (84.0)		667 (69.6)		699 (72.9)		888 (92.6)		750 (78.2)		768 (80.1)		129 (13.5)		719 (75.0)		700 (73.0)	
**Income terciles**				**				***				***		***				**
1st	377 (83.2)		279 (61.6)		334 (73.7)		395 (87.2)		341 (75.3)		277 (61.1)		25 (5.5)		337 (74.4)		303 (66.9)	
2nd and 3rd	738 (84.9)		602 (69.3)		646 (74.3)		812 (93.4)		684 (78.7)		700 (80.6)		120 (13.8)		656 (75.5)		644 (74.1)	
**Household size**																		
≤ 6	668 (84.1)		542 (68.3)		582 (73.3)		723 (91.1)		626 (78.8)		583 (73.4)		95 (12.0)		592 (74.6)		577 (72.7)	
≥ 7	450 (84.4)		341 (64.0)		400 (75.0)		488 (91.6)		403 (75.6)		397 (74.5)		50 (9.4)		404 (75.8)		372 (69.8)	
**Slum household**								***				***		*				
No	597 (84.2)		485 (68.4)		527 (74.3)		671 (94.6)		550 (77.6)		557 (78.6)		89 (12.6)		527 (74.3)		515 (72.6)	
Yes	517 (84.5)		396 (64.7)		452 (73.9)		535 (87.4)		474 (77.5)		419 (68.5)		56 (9.2)		465 (76.0)		431 (70.4)	

Note.

* p-value < .05

** p-value < .01

*** p-value < .001

Only around 10% of all respondents knew a relative or friend who had the disease or died. That proportion was lower among those with low education, low income, and dwelling in slums. However, three-quarters of all participants agreed that such situations should not be kept secret and more than 85% would accept to declare the infection of a relative or friend to the COVID-19 response team (Table 5).

**Table 5:** Acceptability of COVID-19 measures according to baseline characteristics.

	Declaring the infection of a close person to the response team is acceptable		Lockdown measures are acceptable for the community		Partial lockdown in Kinshasa is acceptable		Total lockdown in Kinshasa is acceptable		Mandatory facial mask in public spaces is acceptable		Closure of schools and universities is acceptable		Closure of churches is acceptable		Maintaining minimum services for essential workers is acceptable		Restrictions in public transports is acceptable		Best acceptance level	
	n (%)	p	n (%)	p	n (%)	p	n (%)	p	n (%)	p	n (%)	p	n (%)	p	n (%)	p	n (%)	p	n (%)	p
**Gender**												**		***		**		*		
Male	528 (88.0)		102 (17.1)		152 (25.3)		136 (22.7)		451 (75.2)		335 (55.8)		300 (50.0)		291 (48.5)		423 (70.5)		139 (23.2)	
Female	638 (87.9)		110 (15.2)		170 (23.4)		140 (19.3)		542 (74.7)		336 (46.3)		285 (39.3)		292 (40.2)		475 (65.4)		139 (19.1)	
**Age**						**		*				***		**		**				***
≤ 24	106 (84.8)		14 (11.3)		22 (17.6)		15 (12.0)		96 (76.8)		41 (32.8)		41 (32.8)		43 (34.4)		83 (66.4)		14 (11.2)	
25-35	300 (88.5)		53 (15.6)		61 (18.0)		66 (19.5)		242 (71.4)		187 (55.2)		155 (45.7)		147 (43.4)		238 (70.2)		62 (18.3)	
36-49	365 (87.3)		73 (17.6)		110 (26.3)		94 (22.5)		310 (74.2)		208 (49.8)		176 (42.1)		174 (41.6)		281 (67.2)		90 (21.5)	
50-64	260 (90.6)		42 (14.6)		78 (27.2)		59 (20.6)		223 (77.7)		149 (51.9)		136 (47.4)		143 (49.8)		185 (64.5)		65 (22.6)	
≥ 65	104 (87.4)		20 (16.8)		40 (33.6)		35 (29.4)		96 (80.7)		75 (63.0)		66 (55.5)		66 (55.5)		90 (75.6)		41 (34.5)	
**Religion of household head**								*						**				*		*
Catholic	348 (88.3)		63 (16.0)		100 (25.4)		101 (25.6)		299 (75.9)		214 (54.3)		202 (51.8)		187 (47.5)		281 (71.3)		100 (25.4)	
Protestant	177 (85.1)		35 (16.9)		48 (23.1)		39 (18.8)		153 (73.6)		103 (49.5)		83 (39.9)		84 (40.4)		123 (59.1)		34 (16.3)	
Revival Church	445 (88.6)		82 (16.4)		124 (24.7)		99 (19.7)		387 (77.1)		239 (47.6)		200 (39.8)		209 (41.6)		343 (68.3)		104 (20.7)	
Others	196 (87.9)		32 (14.4)		51 (22.9)		37 (16.6)		154 (69.1)		116 (52.0)		100 (44.8)		103 (46.2)		151 (67.7)		40 (17.9)	
**Education level**				***		**										*				
Low	328 (89.1)		83 (22.6)		108 (29.3)		68 (18.5)		273 (74.2)		172 (46.7)		137 (37.2)		142 (38.6)		244 (66.3)		80 (21.7)	
Medium and high	838 (87.4)		129 (13.5)		215 (22.4)		208 (21.7)		720 (75.1)		500 (52.1)		448 (46.7)		441 (46.0)		654 (68.2)		198 (20.6)	
**Income terciles**				**				**				**		***		***		***		
1st	398 (87.9)		94 (20.9)		115 (25.4)		74 (16.3)		327 (72.2)		206 (45.5)		167 (36.9)		170 (37.5)		274 (60.5)		90 (19.9)	
2nd and 3rd	765 (88.0)		117 (13.5)		207 (23.8)		200 (23.0)		662 (76.2)		464 (53.4)		415 (47.8)		410 (47.2)		620 (71.3)		186 (21.4)	
**Household size**						*														
≤ 6	707 (89.0)		124 (15.7)		178 (22.4)		166 (20.9)		590 (74.3)		398 (50.1)		357 (45.0)		358 (45.1)		546 (68.8)		167 (21.0)	
≥ 7	459 (86.1)		88 (16.5)		145 (27.2)		110 (20.6)		403 (75.6)		274 (51.4)		228 (42.8)		225 (42.2)		352 (66.0)		111 (20.8)	
**Slum household**				**								**		*				*		
No	628 (88.6)		95 (13.4)		168 (23.7)		156 (22.0)		538 (75.9)		383 (54.0)		335 (47.2)		319 (45.0)		498 (70.2)		153 (21.6)	
Yes	534 (87.3)		116 (19.1)		154 (25.2)		117 (19.1)		450 (73.5)		286 (46.7)		246 (40.2)		260 (42.5)		395 (64.5)		122 (19.9)	

Note.

* p-value < .05

** p-value < .01

*** p-value < .001

A minority of participants found lockdown measures acceptable, be it partial or total. In contrast, three-quarters found mandatory facial mask-wearing in public acceptable. Around half of all respondents found the closure of schools and universities acceptable and slightly less for churches. Around four out of ten respondents found it acceptable to maintain a minimum of public services for essential workers. Around two-thirds reported that the restricted number of passengers on public transportation was acceptable. Overall, respondents with the lowest income, younger age groups, and to a lesser degree those from protestant and Revival Church denominations reported a significantly lower level of acceptability compared to the better-off and Catholics. Only younger age groups and protestant denominations remained significant in regression analyses.

### Practices

A majority of respondents (86%) reported regular hand cleaning and mask-wearing followed by physical distancing (72%) (Table 6). Although well observed by more than eight participants out of ten, mask-wearing was the most challenging measure to apply. Avoiding face touching with uncleaned hands (35%), trying not to cough in public (42%), cancellation of events with family and friends (44%), and going less regularly to the market or using public transport (48%) were among the least reported practiced measures. Second and third income tercile respondents were found to have the highest level of best practices compared to first tercile respondents, a finding unchanged in regression analyses. The governmental COVID-19 measures negatively impacted the income-generating activities of around three-quarters of respondents across all three Health Zones (S1 Table). However, this impact was significantly more substantial for Mont Ngafula residents who were more likely to rely on small independent businesses.

**Table 6:** Practices of COVID-19 non-pharmacological interventions according to baseline characteristics.

	Cancelled or postponed external meetings		Reduced use of public transportation		Reduced shopping frequency		Reduced exposure to closed spaces		Avoidance of public gathering		Coughing etiquette		Physical distancing		Regular hand cleaning		Face mask		No face touching with uncleaned hands		No handshake or kissing		Discussed preventive measures with close ones		Best practice level	
	n (%)	p	n (%)	p	n (%)	p	n (%)	p	n (%)	p	n (%)	p	n (%)	p	n (%)	p	n (%)	p	n (%)	p	n (%)	p	n (%)	p	n (%)	p
**Gender**						***				**				*												
Male	316 (52.8)		328 (54.8)		230 (38.5)		330 (55.2)		415 (69.4)		251 (42.0)		452 (75.6)		521 (87.1)		521 (87.1)		212 (35.5)		413 (69.1)		233 (39.0)		226 (37.7)	
Female	367 (50.7)		374 (51.7)		375 (51.8)		377 (52.1)		438 (60.5)		307 (42.4)		503 (69.5)		616 (85.1)		619 (85.5)		254 (35.1)		488 (67.4)		276 (38.1)		284 (39.1)	
**Age**										*				**				*								
≤ 24	58 (46.8)		56 (45.2)		46 (37.1)		63 (50.8)		65 (52.4)		51 (41.1)		73 (58.9)		107 (86.3)		101 (81.5)		41 (33.1)		77 (62.1)		36 (29.0)		40 (32.0)	
25-35	178 (52.5)		179 (52.8)		165 (48.7)		186 (54.9)		222 (65.5)		147 (43.4)		238 (70.2)		285 (84.1)		293 (86.4)		128 (37.8)		237 (69.9)		136 (40.1)		139 (41.0)	
36-49	222 (53.5)		225 (54.2)		191 (46.0)		226 (54.5)		265 (63.9)		181 (43.6)		311 (74.9)		355 (85.5)		356 (85.8)		146 (35.2)		289 (69.6)		166 (40.0)		166 (39.7)	
50-64	152 (53.0)		156 (54.4)		129 (44.9)		156 (54.4)		188 (65.5)		124 (43.2)		224 (78.0)		259 (90.2)		261 (90.9)		107 (37.3)		191 (66.6)		114 (39.7)		114 (39.7)	
≥ 65	60 (50.4)		69 (58.0)		54 (45.4)		58 (48.7)		85 (71.4)		43 (36.1)		82 (68.9)		95 (79.8)		95 (79.8)		37 (31.1)		82 (68.9)		45 (37.8)		40 (33.6)	
**Religion of household head**				**						*				***												
Catholic	220 (55.8)		242 (61.4)		195 (49.5)		228 (57.9)		277 (70.3)		180 (45.7)		311 (78.9)		346 (87.8)		346 (87.8)		156 (39.6)		282 (71.6)		163 (41.4)		165 (41.9)	
Protestant	99 (47.8)		104 (50.2)		87 (42.0)		113 (54.6)		135 (65.2)		87 (42.0)		163 (78.7)		185 (89.4)		179 (86.5)		68 (32.9)		146 (70.5)		72 (34.8)		78 (37.5)	
Revival Church	253 (50.6)		247 (49.4)		218 (43.6)		253 (50.6)		299 (59.8)		197 (39.4)		331 (66.2)		422 (84.4)		435 (87.0)		173 (34.6)		327 (65.4)		184 (36.8)		183 (36.5)	
Others	111 (50.0)		109 (49.1)		105 (47.3)		114 (51.4)		142 (64.0)		94 (42.3)		150 (67.6)		184 (82.9)		181 (81.5)		69 (31.1)		146 (65.8)		90 (40.5)		84 (37.7)	
**Education level**		***		**				***		**		*		***		***		*		**		**		*		*
Low	148 (40.2)		167 (45.4)		162 (44.0)		164 (44.6)		216 (58.7)		136 (37.0)		235 (63.9)		293 (79.6)		304 (82.6)		104 (28.3)		225 (61.1)		124 (33.7)		124 (33.7)	
Medium and high	535 (56.0)		435 (56.0)		443 (46.4)		544 (57.0)		637 (66.7)		422 (44.2)		720 (75.4)		844 (88.4)		837 (87.6)		362 (37.9)		676 (70.8)		385 (40.3)		386 (40.3)	
**Income terciles**		***		***		**		***		***		***		***		***		***		*		**		**		***
1st	189 (42.0)		198 (44.0)		184 (40.9)		208 (46.2)		245 (54.4)		158 (35.1)		288 (64.0)		362 (80.4)		357 (79.3)		138 (30.7)		283 (62.9)		150 (33.3)		141 (31.1)	
2nd and 3rd	490 (56.5)		502 (57.8)		421 (48.5)		498 (57.4)		607 (69.9)		398 (45.9)		664 (76.5)		771 (88.8)		781 (90.0)		326 (37.6)		616 (71.0)		357 (41.1)		369 (42.5)	
**Household size**				*				*																		
≤ 6	406 (51.4)		437 (55.3)		371 (47.0)		445 (56.3)		520 (65.8)		341 (43.2)		580 (73.4)		684 (86.6)		684 (86.6)		293 (37.1)		543 (68.7)		315 (39.9)		318 (40.1)	
≥ 7	277 (52.0)		265 (49.7)		234 (43.9)		263 (49.3)		333 (62.5		217 (40.7)		375 (70.4)		453 (85.0)		509 (85.7)		173 (32.5)		358 (67.2)		194 (36.4)		192 (36.0)	
**Slum household**														*		**		*								
No	379 (53.5)		386 (54.4)		321 (45.3)		388 (54.7)		470 (66.3)		306 (43.2)		531 (74.9)		629 (88.7)		628 (88.6)		262 (37.0)		494 (69.7)		257 (36.2)		273 (38.5)	
Yes	300 (49.3)		313 (51.5)		284 (46.7)		317 (52.1)		381 (62.7)		250 (41.1)		420 (69.1)		503 (82.7)		509 (83.7)		201 (33.1)		404 (66.4)		250 (41.1)		237 (38.7)	

Note.

* p-value < .05

** p-value < .01

*** p-value < .001

## Discussion

To our knowledge, this was the first COVID-19 KAP survey in Kinshasa and the second one in DRC. It was conducted in three Health Zones of Kinshasa three months after strict governmental measures were declared. The survey worryingly shows that around half of the participants had low knowledge scores about COVID-19 transmission, signs and symptoms, and preventive measures. A third of the participants had medium knowledge scores. However, a majority believed in the seriousness of the disease and the effectiveness of preventive measures, although lockdown measures and restrictions in public gatherings were not widely accepted. A majority found wearing masks acceptable and reported doing so regularly along with hand cleaning. Participants who were the poorest, younger, and non-Catholic were overall markedly less knowledgeable and had lower levels of health-protective attitudes, acceptance, and practices compared to others. The education level and household size did not have an influence. So was gender, except that female participants showed less enabling attitudes and, to a lesser extent, practices toward COVID-19 prevention measures than men.

The knowledge level shown in this study echoes the low level of correct knowledge evidenced by the other DRC study [10]. However, it contrasts with findings from Uganda, Nigeria, and Sudan, where participants were found to be knowledgeable or highly knowledgeable about COVID-19 [7–9]. This may be explained by differences in the sampling, which focused, in Uganda, on highly-educated respondents and, in Nigeria and Sudan, on those with access to the Internet to participate in the online survey—a proxy for higher education and wealth levels. In contrast, people with a low educational level and the lowest income tercile constituted approximately a third of our sample, and slightly less than half of the interviewed households were in informal settlements.

In our survey, lockdown measures and restrictions in public gatherings were found to be unpopular—respected only by around half of the respondents, which aligns with research findings from Uganda [7]. An explanation may be that the informal economy predominates in Kinshasa, especially in slum areas, and public interactions in vending and shopping venues or public transports were deemed essential for income generation and household survival [14]. Due to their dominant role in caring for the households, women and girls in fragile contexts, such as in Kinshasa and the DRC, may encounter more difficulties in complying with recommended preventive measures, especially confinement and avoidance of public activities [15]. While limitations of public interactions necessary for safeguarding livelihoods are challenging to respect, a large proportion of respondents reportedly adhered to the more feasible measures of cleaning hands regularly and wearing masks as part of transmission mitigation strategies, as reported in other Sub-Saharan African countries [16]. The compliance to facemasks is surprising given the potential discomfort due to the heat and humidity of Kinshasa’s tropical climate. This, however, illustrates the societal readiness to enact—as feasibly as possible—the perceived urgency and threats of COVID-19.

### Strengths and limitations

Our study had several limitations linked mostly to constrained resources. First, the study covered only three Health Zones out of 35. However, the selected Health Zones were illustrative of the diverse socio-demographic profiles in Kinshasa and included the more impoverished area of Mont Ngafula. Second, this was a unique situational snapshot during the initial emergency state imposed in the DRC. Additional studies are needed to monitor the evolution of the population KAP over time and in response to deconfinement recommendations, new outbreak waves, and other governmental containment measures.

One of the study’s key strengths lies in the fact that the study showcased how public health Master’s students could play a role in COVID-19 public health response beyond community health education, testing, or contact tracing [17]. A large group of properly equipped students was deployed to collect data face-to-face, i.e., outdoor at the doorstep of respondents combined with adequate distancing. Such an approach aligned with the evidence about the minimal outdoor transmission risks of SARS-CoV-2, the virus causing COVID-19 [18]. Additionally, the door-to-door approach ensured a systematic household data collection that did not rely on online surveys and, therefore, enhanced representativeness and reduced participation bias. None of the 85 students reported COVID-19 signs and symptoms as a result of their fieldwork, illustrating the safety and feasibility of such a data collection strategy.

### Implications for policy, practice, and research

The results of this study are essential to inform ongoing and future efforts focusing on enhancing the societal readiness to comply with pandemic prevention and control measures. Our study has highlighted the importance of and need for clear, consistent, and updated public health information to improve the COVID-19-related KAP of the population. Governmental and non-governmental institutions and partners should redouble efforts in crafting adequately formulated and impactful messages. Such efforts should engage community representatives, including women and those found to have markedly lower knowledge and practice levels, i.e., groups belonging to the lowest income tercile, non-Catholic denominations, and younger age cohorts. Further studies are needed to understand the barriers and enablers related to acquiring, applying, and maintaining the optimal KAP level in the specific contexts and priority groups found in Kinshasa, other urban areas in the DRC, and beyond.

## Supporting information

S1 Table Economic impact of COVID-19 prevention measures. (DOCX)
