# Supplementary material for: The emergence of COVID-19 in the Democratic Republic of Congo: Community knowledge, attitudes, and practices in Kinshasa
Source: PLoS One. 2022 Jun 21;17(6):e0265538. doi: 10.1371/journal.pone.0265538 (PMC9212135; doi:10.1371/journal.pone.0265538)
Supplement: S1 Table — (DOCX) [file pone.0265538.s001.docx]

**S1 Table. Economic impact of COVID-19 prevention measures**

|  | Lemba (n=436) | Matete (n=428) | Mont Ngafula (n=459) | Total (n=1323) | p |
| --- | --- | --- | --- | --- | --- |
|  | % | % | % | % |  |
| **Do you do anything to earn money?** |  | | |  | 0,537 |
| Yes | 60.3 | 56.8 | 61.4 | 59.6 |  |
| No | 39.4 | 43.0 | 38.6 | 40.3 |  |
| No answer | 0.2 | 0.2 | 0.0 | 0.2 |  |
| **Activities** |  | | | | |
| Small business | 59.3 | 70.0 | 69.1 | 66.1 | 0,017 |
| Works in a bar | 3.4 | 4.9 | 1.1 | 3.0 | 0,033 |
| Works in a restaurant | 3.0 | 2.1 | 2.8 | 2.7 | 0,77 |
| Domestic | 1.5 | 1.2 | 3.2 | 2.0 | 0,22 |
| Taximan | 4.6 | 3.3 | 5.3 | 4.4 | 0,528 |
| Has a trade | 26.2 | 18.5 | 19.9 | 21.6 | 0,241 |
| Porter | 2.7 | 1.2 | 3.5 | 2.5 | 0,241 |
| Paymaster | 1.5 | 2.1 | 2.8 | 2.2 | 0,567 |
| **Do the measures taken by the government to combat COVID-19 prevent you from carrying out your activities to earn money?** | 0,001 | | | | |
| Yes | 75.7 | 70.3 | 82.4 | 76.3 |  |
| No | 19.5 | 25.9 | 14.2 | 19.7 |  |
| Don't know | 0.7 | 1.2 | 1.3 | 1.1 |  |
| No answer | 4.1 | 2.6 | 2.2 | 2.9 |  |
| **To what extent would you say these activities are disrupted?** |  | | |  | < 0,001 |
| Disrupted a lot | 81.8 | 84.4 | 85.7 | 84.0 |  |
| Disrupted a little | 12.8 | 15.3 | 13.2 | 13.7 |  |
| Not at all disrupted | 5.3 | 0.3 | 1.1 | 2.3 |  |
